# Obesity and diabetes as comorbidities for COVID-19: Underlying mechanisms and the role of viral–bacterial interactions

**DOI:** 10.7554/eLife.61330

**Published:** 2020-09-15

**Authors:** Ilja L Kruglikov, Manasi Shah, Philipp E Scherer

**Affiliations:** 1 Scientific Department, Wellcomet GmbHKarlsruheGermany; 2 Division of Endocrinology, University of Texas Southwestern Medical CenterDallasUnited States; 3 Touchstone Diabetes Center, Department of Internal Medicine, University of Texas Southwestern Medical CenterDallasUnited States; 4 Department of Cell Biology, University of Texas Southwestern Medical CenterDallasUnited States; Icahn School of Medicine at Mount SinaiUnited States; Maine Medical Center Research InstituteUnited States

**Keywords:** adipocyte, bacterial response, viral response, COVID-19, ACE2

## Abstract

Obesity and diabetes are established comorbidities for COVID-19. Adipose tissue demonstrates high expression of ACE2 which SARS- CoV-2 exploits to enter host cells. This makes adipose tissue a reservoir for SARS-CoV-2 viruses and thus increases the integral viral load. Acute viral infection results in ACE2 downregulation. This relative deficiency can lead to disturbances in other systems controlled by ACE2, including the renin-angiotensin system. This will be further increased in the case of pre-conditions with already compromised functioning of these systems, such as in patients with obesity and diabetes. Here, we propose that interactions of virally-induced ACE2 deficiency with obesity and/or diabetes leads to a synergistic further impairment of endothelial and gut barrier function. The appearance of bacteria and/or their products in the lungs of obese and diabetic patients promotes interactions between viral and bacterial pathogens, resulting in a more severe lung injury in COVID-19.

## Introduction

Coronavirus disease-2019 (COVID-2019), caused by the highly pathogenic virus SARS-CoV-2, demonstrates very heterogenous clinical severity, ranging from asymptomatic to devastating forms connected with the development of severe acute respiratory syndrome (SARS) accompanied by extensive pulmonary fibrosis (PF). There is rapidly emerging evidence highlighting obesity and type 2 diabetes (T2D) as comorbidities of SARS development in COVID-19 (Drucker, 2020; Fk et al., 2020; Muniyappa and Gubbi, 2020; Orioli et al., 2020). Clinical studies conducted in different countries demonstrated that obesity and T2D are linked to severe forms of COVID-19 in all ethnic groups. A prospective cohort study on 2741 patients hospitalized in the US health care system revealed that obesity was one of the most important factors associated with hospitalization and critical illness (Petrilli et al., 2020). Another US study on 5700 patients hospitalized with severe forms of COVID-19 reported that many of them had either obesity (41%) or T2D (33%) (Richardson et al., 2020). According to results obtained in China, individuals with obesity compared to patients with normal weight demonstrate significantly more severe forms of COVID-19 (Cai et al., 2020). A meta-analysis based on 33 studies revealed that T2D is associated with mortality and severity of COVID-19 with pooled odds ratios of 1.90 and 2.75, respectively (Kumar et al., 2020). A UK study with 6142 patients indicated that diabetes is an independent prognostic factor in the COVID-19 critical care (Dennis et al., 2020). A retrospective study on 1158 patients hospitalized in Kuwait revealed that patients with morbid obesity and T2D were much more likely to be admitted to the intensive care unit, demonstrating odds ratios of 5.18 and 9.38, respectively (Al-Sabah et al., 2020). Statistically significant correlations were found between the officially reported obesity prevalence and the corresponding number of total deaths of patients with COVID-19 in a number of different countries (Ekiz and Pazarlı, 2020). A strong negative correlation was found between age and BMI in 265 patients admitted to an intensive care unit (ICU), and it was concluded that obesity can shift severe forms of COVID-19 to a younger age (Kass et al., 2020). A single-center retrospective study from Germany based on computed tomography (CT) measurements of visceral and subcutaneous adipose tissue in 30 COVID-19 patients (13 of which had severe forms of disease), revealed that an increase of visceral fat area by one square decimeter was associated with 22.5-fold increased risk to be admitted to ICU and 16.1-fold increased risk for mechanical ventilation (Petersen et al., 2020). Also relevant to this discussion, SARS-CoV-2 clearance is delayed in patients with diabetes (Chen et al., 2020a; Chen et al., 2020b), and T2D as a single comorbidity negatively impacts the severity of COVID-19 (Guo et al., 2020). Additionally, a multi-center retrospective study demonstrated that the high fasting blood glucose is an independent predictor for mortality in patients with COVID-19 without previous diagnosis of diabetes (Wang et al., 2020).

Whereas several pathophysiological mechanisms connecting obesity and diabetes with more pronounced severity of COVID-19 were proposed by different authors, the detailed underlying connections with these comorbidities remain largely unknown and are certainly not yet mechanistically validated in a clinical setting. In the obese and diabetic state, adipose tissue (AT) is compromised and can directly or indirectly be involved in interactions with SARS-CoV-2 at several levels. In the case of direct interactions with the virus, AT, demonstrating higher expression of ACE2 (especially in visceral depots) compared to the lungs (Kruglikov and Scherer, 2020; Al-Benna, 2020), can serve as a big reservoir for viral infections (Kruglikov and Scherer, 2020). A recent in vitro study (Institute of Biology, University of Campinas, Brazil) confirms that the SARS-CoV-2 virus can infect adipocytes where the virus can persist for extended periods of time (https://agencia.fapesp.br/adipose-tissue-may-be-a-reservoir-for-sars-cov-2-brazilian-researchers-suggest/33729/). The virus can also profoundly alter the fate of adipocytes in adipose tissue or adipocyte-like cells in the lungs (Kruglikov and Scherer, 2020). Additionally, SARS-CoV-2 can upregulate genes associated with lipid metabolism in lung epithelial cells, among others the genes involved in regulation of leptin (Al Heialy et al., 2020). This means that SARS-CoV-2 infections modulate lipid metabolism in a similar fashion as observed in the obese or diabetic state.

In the case of indirect interactions, AT can be a source of angiotensin-converting enzyme 2 (ACE2), which is the functional receptor that SARS-CoV/CoV-2 exploits to enter host cells. AT has its own renin-angiotensin system (RAS) that acts in its local microenvironment. Adipocytes express ACE2, and this expression increases during adipogenic differentiation (Gupte et al., 2008) and is further upregulated in the obese state (Zhang et al., 2014; Patel et al., 2016). ACE2 may be shed from AT into circulation and can be deposited in the lungs, thereby modifying pulmonary susceptibility to SAR-CoV-2 infection. This mechanism could be a compensation for significantly increased plasma levels of angiotensin II (Ang II), which is another RAS member triggering vasoconstrictive responses; such Ang II increases were observed in patients with severe COVID-19 manifestations, in which the measured Ang II levels correlated with the severity of lung injury (Liu et al., 2020a). Dysregulation of RAS was even proposed to be the main reason for comorbidity between T2D and severity of COVID-19 (Obukhov et al., 2020).

While generally plausible, these mechanisms, however, do not clearly explain the predominant presence of more severe respiratory forms of COVID-19 in patients with obesity and T2D. The main problem is that this phenomenon is not specific for SARS-CoV/CoV2, and obesity was generally associated with a higher risk of severe viral infections (Huttunen and Syrjänen, 2013). Adiposity was also identified as an independent risk factor in H1N1 swine flu: over 60% of all mortality cases caused by this infection in California happened in obese individuals with odds ratios of 2.8 and 4.2 for patients with BMI >40 and 45, respectively (Louie et al., 2011). A cohort study performed over 12 influenza seasons in Canada revealed that obese individuals with BMI >30 are independently associated with an increased risk of respiratory hospitalizations (Kwong et al., 2011). Similar results were obtained in mice with high fat diet-induced obesity, infected with influenza viruses: these animals demonstrated significantly higher mortality and more severe lung pathology than their lean counterparts (Smith et al., 2007). Of note, influenza viruses bind viral hemagglutinin to sialylated glycans on the plasma membrane, which they use as receptors to infect the host cells. Sialic receptors were incidentally proposed to be an alternative pathway for coronaviruses to enter the host cells (Tortorici et al., 2019).

Obesity and T2D are very prominent, but not the only known comorbidities in COVID-19. Increased risk for severe forms of COVID-19 were also reported in aged individuals and in patients with cardiovascular diseases (CVD) (Mehra et al., 2020) as well as in some ethnic groups. For the general UK population, 34% of COVID-19 patients admitted to ICU were in the Black, Asian and minority ethnic groups, although this group constitutes just 17% of the UK population (Barsoum, 2020). Such prevalence cannot be explained just by cardiometabolic, socio-economic or behavioral factors (Raisi-Estabragh et al., 2020).

**Figure 1.**
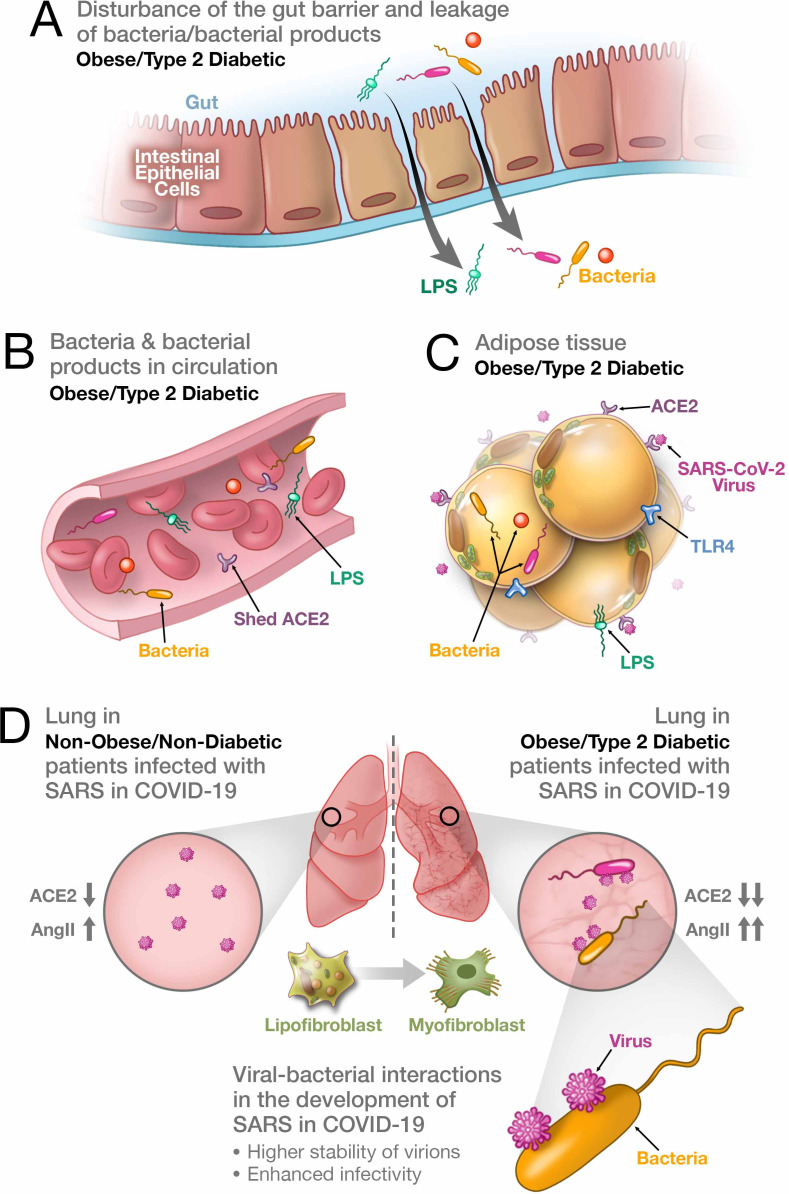
Synergistic interaction between SARS-CoV-2 and bacteria/bacterial products as a possible reason for more severe forms of COVID-19 in patients with obesity and T2D. (**A**) Metabolic dysregulation in obese/T2D patients provides the conditions for disturbance of the gut barrier and leakage of bacteria/bacterial products into the circulation. This dysregulation can be additionally enhanced through viral-induced disbalance in a local renin-angiotensin system (RAS). (**B**) Leakage of bacteria and bacterial products into the circulation provides their system-wide dissemination. (**C**) In the setting of a synergistic effect of viral–bacterial interactions, some bacterial products can trigger an intense response in the adipose tissue. In obesity and T2D, bacteria and bacterial DNA have been found as long-term constituents in different fat depots. (**D**) Increase in circulating LPS will lead to the accumulation of endotoxins in the lung, causing progressive pulmonary inflammation and vascular complications. Viral–bacterial interactions lead to hypercytokinemia – the dysproportional increase in the expression of pro-inflammatory cytokines, which is much higher than what an individual exposure to either viral or bacterial agents can achieve. This also leads to higher stability of virions and enhanced infectivity of SARS-CoV-2. A local synergistic pulmonary ACE2 deficiency develops a disbalance between vasodilating and vasoconstricting RAS agents resulting in inflammation. This interaction can also cause enhanced trans-differentiation of lipofibroblasts into myofibroblasts in obese and T2D patients causing the pulmonary fibrosis.

Whereas the appearance of these comorbidities can have distinct causal explanations, it is more straightforward to assume that all of them have some common underlying pathophysiological mechanism. Below, we critically revisit the factors and pathophysiological pathways that connect obesity and diabetes to the relative severity of SARS in COVID-19, and that are at the same time involved in other comorbidities observed in this disease. We also propose a new hypothesis (Figure 1), which, from our perspective, explains which pathophysiological mechanism may be commonly involved.

## Paradoxical effect of obesity and diabetes on different pulmonary conditions

The established comorbidities - obesity and T2D - that are associated with severe outcomes with SARS in COVID-19 appear to contradict the ‘obesity paradox’, a phenomenon epidemiologically well-established for a number of other diseases. Many studies have determined that patients with obesity and T2D have decreased in-hospital mortality rates connected with multiple pulmonary conditions. Survival of patients and the development of severe forms of chronic obstructive pulmonary disease, characterized by poor gas exchange and strong inflammation, demonstrated statistically significant negative correlations with their BMI values (Landbo et al., 1999; Chittal et al., 2015). Hospitalized obese patients with pulmonary embolism exhibited lower mortality compared to the reference group with odds ratios between 0.56 and 0.63 for different types of obesity (Keller et al., 2019). T2D patients develop acute lung injury (ALI) a lot less frequently than their non-diabetic counterparts, with odds ratios for diabetes and ALI in various studies ranging from just 0.33 to 0.58 (Honiden and Gong, 2009). Analysis of records for 18,450 patients with a primary diagnosis of pulmonary arterial hypertension revealed that obese individuals had lower in-hospital mortality than their non-obese counterparts (adjusted odds ratio of 0.66) (Agarwal et al., 2017).

Similar effects were observed in animal models. The application of lipopolysaccharides (LPS), which are the major components of the outer membrane of Gram-negative bacteria, to non-diabetic mice promoted acute pulmonary inflammation (Dreymueller et al., 2012). In contrast, diabetic Zucker rats demonstrated reduced lung injury and mortality caused by LPS-induced ALI (Wright et al., 1999), and diabetic *db/db* mice developed less severe forms of hyperoxia-induced ALI and exhibited better survival rates (Bellmeyer et al., 2007).

On the other hand, mice with high fat diet-induced T2D susceptible to MERS-CoV infection have more severe clinical symptoms and a delayed recovery. These symptoms were primarily connected with a more severe lung pathology (Kulcsar et al., 2019). MERS-CoV shows about 50% genetic identity to SARS-CoV-2 but utilizes dipeptidyl peptidase-4 (DPP4) instead of ACE2 as its cellular receptor (Muniyappa and Gubbi, 2020). Of note, DPP4 inhibitors are widely used in patients with T2D (Drucker, 2020), which points to similarities in pathophysiological pathways affected in viral infections and T2D.

Such paradoxical reactions of patients with obesity and/or T2D to viral and non-viral pathogens raise the question, which factors can influence the pulmonary severity in COVID-19?

## The role of the angiotensin system for the integral viral load of the target tissue

One of the most obvious and widely accepted primary factors defining the severity of SARS-CoV/CoV-2 infection is the initial viral load. The initial viral loads in patients with severe forms of COVID-19 are thought to be much higher than in patients demonstrating milder forms of this disease (Liu et al., 2020b). On the other hand, viral loads in upper respiratory samples of COVID-19 patients were found to be equal in asymptomatic and symptomatic cases, whereas the clearance rates differed significantly between asymptomatic and symptomatic individuals (Zou et al., 2020). Moreover, additional host factors, such as age and active comorbidities prompted significantly higher hazard ratios for SARS-CoV infections than the viral load alone (Chu, 2004). Importantly, the initial load as a single factor cannot explain the additional risk that comorbidities with obesity or T2D bring along for COVID-19.

Another factor defining severity of COVID-19 is the integral viral load determined by transfer of SARS-CoV-2 viruses into the host cells. This parameter is connected to expression of ACE2 in the host cells. SARS-CoV-2 binds to ACE2 and induces its endocytosis, leading to internalization of the virus/ACE2 complex and a downregulation of cell surface ACE2. ACE2 has at least three functions: it negatively regulates RAS, facilitates the transport of amino acids through association with amino acid transporters and also serves as a functional receptor for SARS-CoV/CoV-2. This enzyme is widely expressed in different organs and tissues, including lungs and AT (Kruglikov and Scherer, 2020; Al-Benna, 2020). Expression of ACE2 in the host cells provides the possibility to modify Ang II to Ang-(1-7), which in turn is a vasodilating agent counteracting the vasoconstricting effect of Ang II through its binding to the Mas receptor. Of note, the reduction of the counter-regulatory axis of RAS promotes the development of fibrosis in different organs and tissues (McKinney et al., 2014; Cha et al., 2018).

ACE2 expression is upregulated in obesity, either induced by high fat (Zhang et al., 2014; Patel et al., 2016) or by a diet high in sucrose (Coelho et al., 2010). Enhanced ACE2 expression induced by high fructose feeding may even induce adipogenesis (Hernández-Díazcouder et al., 2019). Moreover, mice subjected to diet-induced obesity exhibit significantly increased *ACE2* gene expression in the lungs (Al Heialy et al., 2020). Elevated ACE2 expression is also found in the diabetic state (Rao et al., 2020). These authors analyzed a large genome-wide association study with almost 900,000 patient records of individuals with T2D and found out that the appearance of T2D is with high probability causally linked to enhanced ACE2 expression. Further analysis of different datasets confirmed a considerable correlation between ACE2 activity and diabetes (Rao et al., 2020).

On the other hand, reduced ACE2 expression is found in the vasculature of both diabetic animals and humans (Gheblawi et al., 2020). Such a reduction can lead to the development of endothelial dysfunction and increased vascular permeability. Indeed, obese patients display vascular endothelial dysfunction resulting from an imbalance in expression of vasodilatory and vasoconstricting agents (Kwaifa et al., 2020). This dysfunction can be additionally increased through virally-mediated reduction of ACE2. The viral-induced internalization of ACE2 leads to a relative ACE2 deficiency in the host cells, which modifies the local RAS activity and is assumed to be especially harmful in patients with a reduction of ACE2 at the baseline (Verdecchia et al., 2020).

Recently, we have demonstrated that the matrix metalloproteinase MMP14 expression levels are strongly increased in the AT of obese mice (Li and Zhao, 2020). Application of LPS substantially reduced the expression level of MMP14 in lungs; moreover, MMP14^-/-^ mice receiving LPS demonstrated 100% mortality connected with severe lung injury (Aguirre et al., 2017). At the same time, MMP14 is strongly involved in amelioration of inflammation induced by endotoxins (Aguirre et al., 2017). Remarkably, ACE2 KO mice exacerbate Ang II-mediated inflammation and myocardial injury via substantial overexpression of MMP2, −9 and −14 (Song et al., 2013), which can strongly degrade the extracellular and perivascular matrix. Experiments with double mutant Akita (murine model for human diabetes)/ACE2 KO mice revealed that the loss of ACE2 leads to impaired vascular function and activation of MMP-2,–9, and −14 (Patel et al., 2012). Importantly, this effect was observed only in double mutant mice, whereby neither Akita mice nor ACE2 KO mice alone demonstrate such changes. At the same time, non-obese ACE2 KO mice manifest just a mild form of SARS-CoV infection and potently reduced pathological changes in the lungs compared to their wild-type counterparts (Kuba et al., 2005), highlighting the important role of ACE2 for the pathophysiological course of COVID-19.

## The role of ACE2 shedding

Different reports state that ACE inhibitors (ACEi) and Ang II receptor blockers (ARBs), both inducing the expression of ACE2, may be beneficial in COVID-19 (Rossi et al., 2020). In contrast, the application of such inhibitors can increase the severity of COVID-19 through enhancement of the number of functional receptors for SARS-CoV-2 on the surface of critical cells. This point is of high practical importance, since a large number of older patients with hypertension, diabetes, chronic kidney disease and heart failure are on these drugs and can thus be subject to an altered trajectory of COVID-19. A recent report found no evidence for increased severity in a cohort of 1200 hospitalized patients with COVID-19, 399 of which were on ACEi/ARBs (Bean and Kraljevic, 2020). Moreover, the odds ratio for a severe outcome in patients on ACEi/ARB adjusted for age and sex was 0.70. Further adjustments for such comorbidities as hypertension, diabetes, chronic kidney disease and ischemic heart disease demonstrated just a modest additional effect for ACEi/ARBs.

Exposure to ACEi’s or ARB’s induces ACE2 expression, but does not influence its shedding from the cell surface. Such shedding is mainly regulated by Disintegrin and Metalloproteinase Domain 17 (ADAM17), which is a tumor necrosis factor (TNF)-converting enzyme. ADAM17 can however also modulate free ACE2 in circulation (Lambert et al., 2005) and is appreciated as a potential therapeutic target for different inflammatory and vascular conditions (Li et al., 2015). Hyperglycemia, typical in T2D, induces a transcriptional upregulation of ADAM17, and increased ADAM17 protein expression is indeed found in both diabetic patients and in diabetic animal models (Li et al., 2015). The postulated mechanistic basis may primarily be the hyperglycemic upregulation of Ang II, which subsequently, through the AT1R receptor, activates ADAM17, thereby inducing the shedding of ACE2.

Ang II levels in circulation of COVID-19 patients were found to be significantly elevated and linearly associated with viral load and lung injury (Liu et al., 2020b). Dysregulation of RAS associated with the reduction of ACE2 increases vascular permeability, edema, fibrosis and severity of pulmonary injury through uncontrolled action of Ang II (Wang et al., 2020). The overexpression of ACE2 as typically found in individuals with obesity and T2D produces a protective effect against Ang II that otherwise would trigger vasoconstriction in the lung. In obese patients with SARS-CoV-2 infection, the virus causes a significant increase of Ang II levels in circulation, which very likely cannot be compensated by enhanced ACE2 expression, thereby leading to a more severe lung injury. In other words, pulmonary diseases in obesity per se and in obesity combined with viral infections, are governed by the overexpression of different parts of RAS. This effect is very likely contributing to the ‘obesity paradox’.

The suppression of ADAM17 may exert a protective effect on COVID-19 (Palau et al., 2020), since ADAM17 plays an important role in enabling SARS-CoV/CoV-2 to enter host cells through the regulation of the fusion of viral particles with cytoplasmic membranes. Indeed, the application of ADAM17 siRNA reduces SARS-CoV infectivity (Haga et al., 2008).

## Host microbiota

The number of bacteria that humans carry is estimated to be over 100 trillion, which outnumbers by far the total quantity of cells in humans (Velmurugan et al., 2020). Under normal conditions, these bacteria have a commensal relationship and can even be involved in innate and adaptive immunity. On the other hand, dysbiosis of gut and lung microbiota is causally involved in the pathogenesis of cardiopulmonary diseases. Such dysbiosis is connected to ACE2 expression: modulation of ACE2 can strongly modify the composition of microbiota by influencing the amino acid transport and production of antimicrobial peptides (Cole-Jeffrey et al., 2015). Moreover, the development of idiopathic pulmonary fibrosis is directly connected with the enhanced presence of *Staphylococcus* and *Streptococcus* bacteria in the lung (Han et al., 2014), and enteric application of probiotics prevents the development of upper respiratory tract infections (Popova et al., 2012).

Gut microbiota are recognized as the major environmental determinants of obesity and T2D, and gut dysbiosis is involved in the pathogenesis of insulin resistance (Kau et al., 2011; Anhê et al., 2020). A number of recent reports described live bacteria and bacterial DNA as long-term constituents in different fat depots in obesity and T2D (Anhê et al., 2020; Gurung et al., 2020; Massier et al., 2020). This may be related to the decreased barrier function in these individuals. Whereas the spectrum of bacteria is different in obesity and T2D, both Gram-negative and Gram-positive bacteria can be found in human adipose tissues (Anhê et al., 2020; Massier et al., 2020). Bacteria, bacterial DNA and bacterial products such as LPS are also detected in circulation of obese and T2D individuals (Sato et al., 2014; Velmurugan et al., 2020; Anhê et al., 2020), which means that patients with these diseases suffer from a system-wide dissemination of bacteria. The relative physiological importance of metabolic endotoxemia – i.e. the translocation of bacteria-derived LPS into circulation - in obesity and T2D as well as in cardiovascular and pulmonary diseases, is a matter of intense investigation (Cani et al., 2007; Neves et al., 2013). Metabolic endotoxemia was connected to the development of low-grade inflammation through activation of toll-like receptors 4 (TLR4) (Neves et al., 2013). Such low-grade systemic inflammation (meta-inflammation) was argued to be an obvious reason for high mortality of patients with COVID-19 having metabolic diseases (Mauvais-Jarvis, 2020). Both viral and bacterial pathogens can activate the TLR4 pathway, and modification of TLR4 signaling is reported to be involved in different viral infections including SARS-CoV (Olejnik et al., 2018) as well as in LPS-induced acute lung injury (Ye and Liu, 2020). Of note, ADAM17 is implicated in shedding of not only ACE2, but also of TLR4 (Yang et al., 2018). Even under normal metabolic conditions, we have shown that TLR4 plays important regulatory roles for metabolism (Holland et al., 2011; Jia et al., 2014; Tao et al., 2017; Jia et al., 2018). It is therefore not surprising that several groups recently drew attention to a possible connection between the gut microbiota in healthy individuals and the potential severity of COVID-19 (Dhar and Mohanty, 2020; Gou et al., 2020).

Microbiota are present not only in gut, but also in other tissues and organs. Some bacteria trigger an intensive response in adipocytes, others seem to use AT as a safe haven, protecting them from the immune system. Experiments looking into the interaction of *Mycobacterium tuberculosis* with primary mouse preadipocytes (3T3-L1 cells) and primary human adipocytes in vitro revealed that this pathogen enters adipocytes and survives inside these cells in a non-replicative state, completely protected from anti-mycobacterial drugs (Neyrolles et al., 2006). *Rickettsia prowazekii* (the causative agent of typhus) can infect and replicate in 3T3-L1 cells rendering adipose tissue into a dormant reservoir for this pathogen (Bechah et al., 2010). *Coxiella burnetii* (the agent of query fever) is found in AT even four months post-infection, when no bacteria are detectable in other tissues, thus making AT a safe haven and a long-term reservoir, prevailing even after apparent full clinical recovery (Bechah et al., 2014). While no direct evidence points to microbiota in AT serving as an additional source for endotoxins, we assume this is very likely.

Similar to the synergistic effects of diabetes and ACE2 deficiency on endothelial function and overexpression of MMPs observed in Akita/ACE2 KO double mutant mice (Patel et al., 2012), synergistic interactions of these two factors are also observed in the modification of gut microbiota and gut barrier function (Duan et al., 2019). Such synergism should lead to a leakage of pathogens and/or their products from the gut and adipose tissue into circulation. For example, the increase in circulating LPS will lead to the accumulation of endotoxins in the lung, causing progressive pulmonary inflammation and vascular complications.

## Interaction of LPS with lipoproteins

Whereas a large part of LPS in circulation can be neutralized through its binding to high density lipoproteins (HDL), resulting in the clearance of LPS through biliary excretion, a smaller part of LPS does not associate with HDL and is responsible for the activation of macrophages and the overproduction of potent inflammatory mediators (Vishnyakova et al., 2003). Both HDL and LPS bind to the scavenger receptors class B type I (SR-BI). These receptors belong to a cholesterol delivery system and are present in different types of cells, including adipocytes and type two alveolar epithelial cells. In fact, the spike protein of SARS-CoV-2 binds to HDL cholesterol (HDL-C), and the severity of the SARS-CoV-2 infection is inversely associated with plasma levels of HDL-C. Surprisingly, antagonists of the HDL receptor SR-BI may inhibit SARS-CoV-2 infection (Wei et al., 2020). Consequently, it may be that patients on statins are more susceptible to severe forms of COVID-19. However, multiple studies in the literature have made the case that individuals on statins may in fact be protected and show a reduced rate of mortality (Zhang et al., 2020).

HDL, non-lipoprotein-bounded LPS and SR-BI were found to be associated with plasma membrane invaginations (caveolae) in different cell types (Vishnyakova et al., 2003). This is especially interesting since different authors reported that cholesterol-rich rafts in plasma membrane are substantially involved in penetration of SARS-CoV virus into the host cells (Meher et al., 2019) and that the application of methyl-β-cyclodextrin (depleting cholesterol from plasma membranes and destroying caveolae) significantly impairs the efficiency of viral entrance (Ren et al., 2008).

Since the pathways involving SR-BI can be exploited both by SARS-CoV2 virus and LPS, this raises the next question concerning the role of interactions between viral and bacterial pathogens in severity of COVID-19.

## Synergistic viral–bacterial interactions and the severity of COVID-19

It is already established that microbiota can directly or indirectly impact the outcome of different viral infections (Neu and Mainou, 2020). Viruses bind to bacteria through LPS (by Gram-negative bacteria) or peptidoglycan (by Gram-positive bacteria), and this binding can provide enhanced attachment of the virus to its receptor on the surface of host cells, thereby enhancing its infectivity (Neu and Mainou, 2020). This effect can sufficiently increase the integral viral load in the lungs. On the other hand, respiratory viruses can promote bacterial pneumonia, thereby altering the microbiota in the upper respiratory tract and promoting bacterial accumulation in the lower respiratory tract (Lee et al., 2016).

Strong synergistic effects of combined coronavirus and bacterial infections on the severity of lung injury was demonstrated in the porcine respiratory coronavirus (PRCV) model (VAN Reeth et al., 2000; Van Gucht et al., 2006). PRCV is common in swine populations and shares several pathogenetic characteristics with SARS-CoV/CoV-2. Combination of PRCV infection with LPS effectively increased severity of SARS: whereas pigs exposed to either PRCV or LPS demonstrated no or only mild symptoms, the combination of PRCV and LPS induced severe SARS in the majority of animals (Van Gucht et al., 2006). Synergistic interactions between these pathogens provided a hypercytokinemia – a dysproportional (about 60 times) increase in the expression of pro-inflammatory cytokines TNF-α and IL-6 in bronchoalveolar lavage fluid, which is also typically seen in severe COVID-19 patients requiring ICU admission (Coperchini et al., 2020). Similar synergistic effects leading to considerable worsening of PRCV infections were observed not only for the interactions of viral pathogens with bacterial products, but also in combined viral–bacterial infections (Opriessnig et al., 2011).

Similar results were obtained for the interactions between influenza virus (pH1N1) and LPS in mice: such interactions resulted in a synergistic increase in TNF-α, IL-1β, and IL-6 levels in lung tissue, promoting a hypercytokinemia and a pulmonary pro-inflammatory immune response (Koch et al., 2018).

LPS induces lung injury through the suppression of ACE2 and the upregulation of Ang II, ACE, and AT1 receptors, thus significantly modulating the whole RAS (Ye and Liu, 2020). There are several factors influencing the endotoxicity of LPS, some of them acting as enhancers (LPS binding protein and CD14), others as suppressors (haptoglobin). All these factors are strongly dysregulated in the PRCV model (Van Gucht et al., 2006). On the other hand, plasma levels of LPS binding protein are increased in obesity and T2D, demonstrating a positive correlation with BMI (Sato et al., 2014); CD14 expression is enhanced in obese humans (Fernández-Real et al., 2011), and the serum levels of haptoglobin are increased both in diabetic rats (Jelena et al., 2013) and in T2D patients (McMillan, 1989). Enhanced expression of haptoglobin correlates with serum viscosity and was causally connected with the appearance of various microangiopathies in T2D patients (Wang et al., 2018). Importantly, micro-thrombosis is very common in COVID-19 pneumonia (Price et al., 2020).

Whereas the gut-lung axis is well established as an important determinant of severity of the pulmonary diseases (Marsland et al., 2015; Budden et al., 2017), the role of metabolic endotoxemia in severity of COVID-19 remains poorly investigated. Nevertheless, several groups have reported significant changes in gut microbiota and in LPS levels in patients with severe forms of COVID-19. ACE2 has a major impact on the composition of gut microbiota. ACE2 modulation by viral infection can significantly influence its content and leakage from the gut; indeed, severe forms of COVID-19 were connected with pronounced gastrointestinal symptoms (Gou et al., 2020). Post-mortem analysis of twenty COVID-19 patients demonstrated that *Enterobacteriaceae spp.*, which are abundant in the human gut and can release a large amount of endotoxin, were commonly present in the lung tissue (Fan et al., 2020). A cross-sectional study of 30 patients with COVID-19, 24 patients with influenza A, and 30 matched healthy controls revealed a significantly higher abundance of opportunistic pathogens in gut microbiota of COVID-19 patients (Gu and Chen, 2020). In a small prospective study on 19 patients with severe pulmonary forms of COVID-19, bacterial DNA and toxins were found in blood samples of almost all individuals, whereas over 40% of them had high, and over 89% had increased endotoxin levels measured with chemiluminescent-based endotoxin activity assay (Sirivongrangson et al., 2020).

Based on these results, we suppose that bacteria and their by-products in circulation can be translocated to the lungs where they interact with viral infections, producing conditions similar to those observed in the PRCV model and thus worsening the severity of COVID-19. Dysfunctional adipose tissue as seen in the context of obesity and T2D may not only be an additional source of these bacterial products, but they are also likely to interact with viral particles with an affinity for the ACE2 receptors on the surface of adipocytes.

## Possible role of viral and bacterial pathogens in pulmonary fibrosis

Lung consolidation, i.e. regions of lung tissue that are filled with liquid instead of air, leading to the development of SARS, is a common complication in COVID-19, and the development of a pronounced pulmonary fibrosis is evident in non-survivors (Zuo et al., 2010). The appearance of pulmonary fibrosis in viral infections is connected with TGF-β overexpression as well as with the suppression of ACE2 (Zuo et al., 2010). Recently we have proposed that these processes can lead to trans-differentiation of pulmonary lipofibroblasts into myofibroblasts, thereby inducing severe forms of pulmonary fibrosis and exerting a negative impact on pulmonary gas exchange (Kruglikov and Scherer, 2020). If a combination of viral and bacterial infections induces more severe forms of COVID-19, we expect the trans-differentiation of lipofibroblasts into myofibroblasts should also be enhanced under conditions of combined exposure of SARS-CoV-2 with LPS.

Lipofibroblasts can trans-differentiate into myofibroblasts under different conditions, including hyperoxia and infection (Torday and Rehan, 2007). This transformation was connected with a deprivation of parathyroid hormone-related protein (PTHrP), secreted by type two alveolar epithelial cells. Application of LPS reduces the expression of PTHrP and enhances the expression of αSMA, which is the marker of bronchopulmonary dysplasia (Torday and Rehan, 2007). Similarly, LPS can lead to a trans-differentiation of pericytes into myofibroblasts in renal fibrosis (Castellano et al., 2019). LPS-stimulated pericytes secrete LPS binding protein and TGF-β and undergo trans-differentiation, even upon TGF-β receptor-blocking. This indicates the involvement of TLR4 signaling (Castellano et al., 2019). Remarkably, the therapeutic application of citrate-based coupled plasma filtration absorption (CPFA) in this study significantly reduced serum levels of TGF-β and LPS binding protein and inhibited trans-differentiation of pericytes, which may be also useful in clinical applications to prevent the trans-differentiation of lipofibroblasts over the course of COVID-19.

Not only bacterial products, but also the intracellular bacteria themselves can induce massive phenotypical changes in the host cells (Niller et al., 2017). Whether such modifications can cause trans-differentiation of adipocytes and/or lipofibroblasts will need to be investigated in future research.

## Application of anti-diabetic drugs in COVID-19 patients

The acute inflammatory state in severe COVID-19 is connected to significant hyperglycemia, and this effect is especially pronounced in patients with diabetes, prediabetes and obesity (Gianchandani et al., 2020). Interestingly, some critically ill COVID-19 patients without diabetes also develop hyperglycemia (Gianchandani et al., 2020). Severe hyperglycemia is even proposed to be an independent predictor of death and morbidity in various infectious diseases, including COVID-19 (Apicella et al., 2020). This renders the glycemic control in these patients an important challenge and begs the question whether the use of anti-diabetics, such as insulin and PPARγ agonists, should be used in COVID-19 patients. Arguments for and against the use of anti-diabetics, such as PPARγ agonists, metformin, SGLT2-inhibitors and GLP-1 receptor agonists were recently discussed in Apicella et al., 2020.

Remarkably, COVID-19 patients on metformin have better clinical outcomes than patients on insulin (Chen et al., 2020a; Chen et al., 2020b). While insulin administration caused the suppression of ACE2 expression in a number of different diabetic mouse models (Roca-Ho et al., 2017), exposure to pioglitazone significantly upregulates ACE2 expression in different organs and tissues in rats (Zhang et al., 2014). Whether this is generally true remains to be seen, as we have little evidence for this phenomenon occurring in mice exposed to PPARγ agonists (our unpublished observations).

It is very likely that beyond the suppression of hyperglycemia and the possible regulation of ACE2 expression, PPARγ agonists can demonstrate also another effect in COVID-19 patients. These drugs can modulate endotoxin levels. Indeed, the use of rosiglitazone in a rat model of bronchopulmonary dysplasia induced by LPS significantly attenuated the negative effects of LPS and reduced lung injury (Lee et al., 2014). A direct reduction of LPS in circulation was reported in a study of 346 patients with T2D, which were for one year either diet-controled or treated with metformin, rosiglitazone, a combination of metformin/rosiglitazone or insulin (Al-Attas et al., 2009). Whereas different PPARγ agonists demonstrated a reduction of LPS levels in the plasma of treated T2D patients, the most pronounced reductions were observed in the group treated with rosiglitazone, whereas the highest LPS values were found in the group treated with insulin.

Additionally, the use of such PPARγ agonists (commonly referred to as thiazolidinediones (TZDs)) can improve the adipogenic phenotype in lipofibroblasts thus reducing the trans-differentiation of these cells into myofibroblasts and theoretically preventing the development of pulmonary fibrosis (Kruglikov and Scherer, 2020).

## Possible roles of viral–bacterial interaction in other comorbidities in COVID-19

As we discussed above, several pathophysiological mechanisms may be involved in development of severe forms of COVID-19 (Table 1). These mechanisms can be roughly divided in two groups: those connected with modulation of ACE2 receptor (thus directly or indirectly influencing the status of the local or generalized renin-angiotensin system) and those providing an interaction between viral infection and pre-existing bacterial conditions in different tissues as well as in circulation.

**Table 1.** Some possible pathophysiological pathways connecting obesity/T2D to severity of COVID-19.

Nr.	Description	Comments
1.	**High integral viral load induced by the local up-regulation of ACE2** Expression of angiotensin-converting enzyme 2 (ACE2), which is the functional receptor that SARS-CoV/CoV-2 exploits to enter host cells, is strongly upregulated in different tissues of patients with obesity and T2D. This can lead to a high integral viral load of these tissues.	**Pro** Non-obese ACE2 KO mice manifest a mild form of SARS-CoV infection and strongly reduced pathological changes in the lungs compared to their wild-type counterparts. **Contra** Comorbidity of obesity/T2D with severity of COVID-19 was observed in viral infections other than SARS-CoV/CoV-2 and thus is not ACE2 specific. ACE inhibitors (ACEi) and Ang II receptor blockers (ARBs), both inducing the expression of ACE2, are thought to be beneficial in COVID-19.
2.	**Shedding of ACE2** Increased shedding of ACE2 from different tissues (including adipose tissue demonstrating high expression of this enzyme in obesity and T2D) induced by ADAM17 leads to re-distribution of ACE2 in the body and its accumulation in the lungs.	**Pro** Hyperglycemia, typical in obesity and T2D, induces increased ADAM17 protein expression. Application of ADAM17 siRNA reduces SARS-CoV infectivity. **Contra** Comorbidity of obesity and T2D with severity of COVID-19 was observed in viral infections other than SARS-CoV/CoV-2 and thus is not ACE2 specific. ADAM17 regulates the fusion of viral particles with cytoplasmic membranes involved in entering of SARS-CoV/CoV-2 into the host cells. Thus, the positive effect of ADAM17 suppression is not in an obvious way connected with a reduced ACE2 shedding.
3.	**Disturbance of the vasodilation-vasoconstriction balance in the RAS system** The pulmonary renin-angiotensin system (RAS) is adapted to the conditions of increased ACE2 expression in obese individuals. Enhanced internalization of the virus/ACE2 complex leads to a quick production of a local pulmonary ACE2 deficiency, thereby disturbing the balance between vasodilating (Ang-(1-7)) and vasoconstricting (Ang II) agents in RAS and inducing the development of inflammation and fibrosis.	**Pro** ACE inhibitors (ACEi) and Ang II receptor blockers (ARBs), both inducing the expression of ACE2, are thought to be beneficial in COVID-19. Levels of vasoconstricting agent Ang II in circulation of COVID-19 patients are significantly elevated and linearly associated with viral load and lung injury. **Contra** Comorbidity of obesity and T2D with severity of COVID-19 was observed in viral infections other than SARS-CoV/CoV-2 and thus is not ACE2 specific. ACE2 deficiency seems to be not a single parameter influencing this effect. ACE2 KO mice exacerbate Ang II-mediated inflammation via overexpression of matrix metalloproteinases MMP2, −9 and −14.
4.	**Compromised endothelial function in obesity and diabetes** The vasculature of obese and diabetic subjects has a reduced baseline ACE2 expression, which leads to a compromised endothelial function and increased vascular permeability. This dysfunction can be further increased through virally-mediated reduction of ACE2. Administration of insulin and other anti-diabetic drugs causes additional suppression of ACE2.	**Pro** ACE inhibitors (ACEi) and Ang II receptor blockers (ARBs), both inducing the expression of ACE2, are thought to be beneficial in COVID-19. Experiments with double mutant Akita (murine model for human diabetes)/ACE2 KO mice revealed that the loss of ACE2 leads to impaired vascular function. This effect was observed only in double mutant mice, whereby neither Akita mice nor ACE2 KO mice alone demonstrate such changes. **Contra** Comorbidity of obesity and T2D with severity of COVID-19 was observed in viral infections other than SARS-CoV/CoV-2 and thus is not ACE2 specific.
5.	**Synergistic viral–bacterial interaction** Binding of viruses to lipopolysaccharides (LPS) can enhance their attachment to receptors on the surface of the host cells, thereby enhancing the infectivity. This effect can synergistically increase the integral viral load in the lungs. On the other hand, respiratory viruses can promote bacterial pneumonia, thereby altering the microbiota in the upper and promoting bacterial accumulation in the lower respiratory tract.	**Pro** Bacteria, bacterial DNA and LPS are present in circulation of obese and T2D individuals, and metabolic endotoxemia is causally connected with obesity, T2D, cardiovascular and pulmonary diseases. The spike protein of SARS-CoV2 binds to high density lipoprotein (HDL) cholesterol, and the severity of the SARS-CoV2 infection is inversely associated with plasma levels of HDL cholesterol. HDL and LPS bind to the scavenger receptor class B type I, which belongs to a cholesterol delivery system and is present in cells such as adipocytes and type two alveolar epithelial cells. Strong synergistic effects of combined coronavirus and bacterial infections on the severity of lung injury was demonstrated in the porcine respiratory coronavirus (PRCV) model: whereas pigs exposed to either PRCV or LPS demonstrated no or only mild symptoms, the combination of PRCV and LPS induced severe SARS and death in the majority of animals. Similar synergistic effects were observed in combined viral–bacterial infections. LPS induces lung injury through the suppression of ACE2 and the upregulation of Ang II, ACE, and AT1 receptors, thus dysregulating RAS before the viral infection. LPS binding protein (an enhancer of LPS endotoxicity) demonstrates a positive correlation with BMI and is significantly elevated in obesity and T2D. Synergistic viral–bacterial interactions seem to be involved in comorbidities of severe COVID-19 beyond obesity and T2D.
6.	**Cellular transformations in lungs** Adipose tissue is generally compromised in obesity and T2D. SARS-CoV/CoV-2 virus additionally modifies adipocytes and adipocyte-like cells causing their differentiation state, which directly modifies the function of the tissue containing these cells. In severe forms of SARS, this may involve the trans-differentiation of pulmonary lipofibroblasts into myofibroblasts.	**Pro** Lipofibroblasts trans-differentiate into myofibroblasts under different conditions, including hyperoxia and infection. This transformation is connected with a deprivation of parathyroid hormone-related protein, secreted by type two alveolar epithelial cells, which is suppressed by LPS. LPS leads to the trans-differentiation of pericytes into myofibroblasts in renal fibrosis. LPS-stimulated pericytes undergo trans-differentiation, even upon TGF-β receptor-blocking, which suggests the involvement of TLR4 signaling. Modification of TLR4 signaling is involved in different viral infections including SARS-CoV. Application of the PPARγ agonist rosiglitazone in a rat model of bronchopulmonary dysplasia induced by LPS significantly attenuates the negative effects of LPS and leads to reduced lung injury.

While all of them can theoretically contribute to the severity of COVID-19, we believe that one of these mechanisms should play the predominant role. Such mechanism must be present not only in obese and diabetic patients, but also in other groups of increased risk in COVID-19, including aged individuals, patients with CVD and some ethnicities. Moreover, taking into account multiple reports underlining the influence of gender and ethnicity on the COVID-19 outcome, this pathway must be also differentially present in males and females as well as in different ethnic groups. Hypothesis concerning the primary relevance of synergistic viral–bacterial interaction in severity of COVID-19 matches all these demands.

Indeed, inflammation induced by bacteria and LPS is involved not only in insulin resistance but also in development of vascular abnormalities; hence, it is not surprising that blood-borne microbiota and circulating microbial metabolites were found to be significantly modified not only in diabetes but also in CVD (Velmurugan et al., 2020). Bacterial translocation from the gut to circulation caused by disruption of the gut barrier function was described both in patients with myocardial infarction and in corresponding mouse models (Zhou et al., 2018). This clearly indicates that viral–bacterial interactions are likely to also be involved in the comorbidity of CVD with COVID-19.

Older patients demonstrate an increased risk for the development of severe forms of COVID-19 (Mehra et al., 2020). Human gut microbiota also demonstrate significant modification along with the host aging (Xu et al., 2019). The plasma levels of LPS and its binding protein were doubled in older individuals (mean age of 73.8) compared to their younger counterparts (mean age of 25.5) (Ghosh et al., 2015). This effect was observed even in lean healthy aged individuals, providing they display pre-conditions for viral–bacterial interactions similar to those in individuals with obesity and/or T2D. Additionally, LPS is a well-known mediator of inflammation, and the levels of adipose-derived IL-6 induced by the same dose of LPS were shown to be significantly higher in older compared to young mice (Starr et al., 2009). Importantly, viral–bacterial interactions can provide synergistic cytokine induction (Van Gucht et al., 2006; Koch et al., 2018), leading to hypercytokinemia (‘cytokine storm’) typical in COVID-19.

Plasma levels of LPS demonstrate also pronounced ethnic and gender variations. Age-adjusted levels of endotoxin were significantly lower in females than in males and significantly varied in different ethnic groups being the highest in South Asians (Miller et al., 2009). This can at least partly explain the high severity of COVID-19 observed in ethnic minorities in UK (Barsoum, 2020).

Whereas clinical arthritis was not revealed as a comorbidity of COVID-19 severity, this question will need additional investigation in the future, especially because some arthritis drugs were applied for treatment of COVID-19. Serum and synovial fluid LPS in osteoarthritis are associated with the presence of activated macrophages in the synovium, indicating that metabolic endotoxemia must be involved in pathogenesis of this disease (Huang et al., 2016). Moreover, oral administration of LPS reactivated antigen-induced arthritis in mice promoting expression of interferon-γ, interleukin-1β, and TNF-α (Yoshino et al., 2005). This may be the reason for remarkably similar pattern of pro-inflammatory cytokines observed in clinical arthritis and COVID-19 (Schett et al., 2020).

Blood microbiota were also associated with such cognitive diseases as Alzheimer and Parkinson (Velmurugan et al., 2020). LPS as well as its fibrin-association LPS were found in blood samples of these patients as well as of patients with T2D and it is known that the presence of LPS can trigger hypercoagulation, causing anomalous blood clotting and eventual development of thromboembolic conditions (de Waal et al., 2018). Viral infections are also associated with coagulation disorders (Goeijenbier et al., 2012), and synergistic viral–bacterial interaction in patients with severe form of COVID-19 can provide a significant worsening of this condition. Indeed, a Dutch study on 184 patients with COVID-19 admitted to ICU revealed that 31% of them had thrombotic complications, which is remarkably high (Klok et al., 2020).

Recently, a study on a mouse model exposed to a refined diet, containing just insoluble but no soluble fibers, demonstrated a significant modification of the gut microbiota independent of the age and gender of the animals (Morrison et al., 2020). Given the possible role of blood microbiota and circulating microbial metabolites in COVID-19, we can assume that refined diets rich in insoluble fibers may play a role in severity of COVID-19. This relationship may deserve to be investigated in future studies.

## Should therapeutic options be revised?

It is beyond the scope of this article to present an in-depth discussion of possible therapeutic options based on the proposed pathophysiological pathways. Assuming these pathways outlined are pathophysiologically relevant, we can think of three therapeutic options, which may be worthwhile to consider from our perspective. First, it may be of clinical value to apply citrate-based coupled plasma filtration adsorption (CPFA) to reduce the levels of TGF-β as well as of bacteria and bacterial products in circulation to avoid their deposition in the lungs. Second, the use of PPARγ agonists may be highly appropriate in this context, since (a) they can actually be potently anti-inflammatory, (b) can reduce hyperglycemia (common in critical COVID-19 patients), (c) can modulate ACE2 expression, (d) reduce LPS levels in circulation, and (e) improve the adipogenic phenotype in lipofibroblasts, thus reducing their trans-differentiation into myofibroblasts. Third, there may be a justification for the development of therapeutic methods that lead to a reduction of hyperoxia in the lung. Hyperoxia can promote trans-differentiation of lipofibroblasts into myofibroblasts as well. Clearly, future endeavors will need to test these proposed therapeutic options and their efficacy.

## Conclusions

There are several established comorbidities for severe forms of SARS in COVID-19, and obesity and diabetes rank at the very top. Pathophysiological pathways involved in these comorbidities include a series of remarkable synergistic interactions. Adipose tissue demonstrates high expression of ACE2 that SARS-CoV-2 exploits to enter host cells and which makes adipose tissue a prime reservoir for SARS-CoV-2 viruses, thus increasing the integral viral load. Acute viral infection results in ACE2 reduction in different organs and tissues. This deficiency can lead to disturbances in two other systems controlled by ACE2: renin-angiotensin system, where ACE2 has the role of a negative regulator, and transport of amino acids, where ACE2 associates with amino acid transporters, enhancing their function. Such disturbances are further increased in the case of pre-conditions with already compromised function of these systems. This is the case in patients with obesity and diabetes characterized by substantially compromised endothelial function and increased vascular permeability. Whereas such interactions are not specific for SARS-CoV-2 and are observed in other viral infections, COVID-19 exposure is more severe, which is mainly due to the high affinity for ACE2 which is in SARS-CoV-2 at least an order of magnitude higher than for SARS-CoV.

We propose that the synergistic interactions of virally-induced ACE2 deficiency with obesity and/or diabetes provide a synergistic impairment of endothelial function and gut barrier function, resulting in the leakage of bacterial pathogens and their products from the gut into circulation. Bacterial DNA and bacterial products can be detected in circulation of obese and T2D individuals, which predisposes patients with these diseases to a system-wide bacterial dissemination. The appearance of bacteria and/or their products in the lungs promotes an additional synergistic interaction between viral and bacterial pathogens - resulting in more severe lung injury in COVID-19 than in the case of a single viral or bacterial infection. This is further supported by the high levels of bacterial products in circulation of patients with severe forms of COVID-19 and the strong modulation of gut microbiota in critically ill COVID-19 patients. Additionally, further experimental support comes from observations demonstrating synergistic viral–bacterial interactions that can lead to the development of a hypercytokinemia (‘cytokine storm’), which is a hallmark of severe COVID-19 cases. The involvement of viral–bacterial interactions can also explain the increased risk of severe COVID-19 in older patients, in patients with cardiovascular diseases, and in some ethnic groups, as previously described in the literature. More comprehensive clinical and experimental research will be needed to further substantiate this interesting pathophysiological mechanism.
